# First Genome Sequences of Two Multidrug-Resistant *Candida haemulonii* var. *vulnera* Isolates From Pediatric Patients With Candidemia

**DOI:** 10.3389/fmicb.2020.01535

**Published:** 2020-07-03

**Authors:** Luiza Souza Rodrigues, Rajesh Kumar Gazara, Hemanoel Passarelli-Araujo, Andressa Eloisa Valengo, Paula Veronesi Marinho Pontes, Rodrigo Nunes-da-Fonseca, Robson Francisco de Souza, Thiago Motta Venancio, Libera Maria Dalla-Costa

**Affiliations:** ^1^Faculdades Pequeno Príncipe, Curitiba, Brazil; ^2^Instituto de Pesquisas Pelé Pequeno Príncipe, Curitiba, Brazil; ^3^Laboratório de Química e Função de Proteínas e Peptídeos, Centro de Biociências e Biotecnologia, Universidade Estadual do Norte Fluminense Darcy Ribeiro, Campos dos Goytacazes, Brazil; ^4^Department of Biotechnology, Indian Institute of Technology Roorkee, Roorkee, India; ^5^Department of Electrical Engineering, Indian Institute of Technology Roorkee, Roorkee, India; ^6^Departamento de Bioquímica e Imunologia, Instituto de Ciências Biológicas, Universidade Federal de Minas Gerais, Belo Horizonte, Brazil; ^7^Instituto de Biodiversidade e Sustentabilidade, Núcleo em Ecologia e Desenvolvimento Sócio-Ambiental de Macaé, Universidade Federal do Rio de Janeiro, Macaé, Brazil; ^8^Laboratório de Estrutura e Evolução de Proteínas, Instituto de Ciências Biomédicas, Universidade de São Paulo, São Paulo, Brazil

**Keywords:** *Candida haemulonii* var. *vulnera*, candidemia, whole-genome sequencing, multidrug-resistance clade, fungal infection

## Abstract

*Candida haemulonii* is a complex formed by *C. haemulonii sensu stricto*, *C. haemulonii* var. *vulnera*, and *C. duobushaemulonii*. Members of this complex are opportunistic pathogens closely related to *C. pseudohaemulonii*, *C. lusitaniae*, and *C. auris*, all members of a multidrug-resistant clade. Complete genome sequences for all members of this group are available in the GenBank database, except for *C. haemulonii* var. *vulnera*. Here, we report the first draft genomes of two *C. haemulonii* var. *vulnera* (isolates K1 and K2) and comparative genome analysis of closely related fungal species. The isolates were biofilm producers and non-susceptible to amphotericin B and fluconazole. The draft genomes comprised 350 and 387 contigs and total genome sizes of 13.21 and 13.26 Mb, with 5,479 and 5,507 protein-coding genes, respectively, allowing the identification of virulence and resistance genes. Comparative analyses of orthologous genes within the multidrug-resistant clade showed a total of 4,015 core clusters, supporting the conservation of 24,654 proteins and 3,849 single-copy gene clusters. *Candida haemulonii* var. *vulnera* shared a larger number of clusters with *C. haemulonii* and *C. auris*; however, more singletons were identified in *C. lusitaniae* and *C. auris*. Additionally, a multiple sequence alignment of Erg11p proteins revealed variants likely involved in reduced susceptibility to azole and polyene antifungal agents. The data presented in this work will, therefore, be of utmost importance for researchers studying the biology of the *C. haemulonii* complex and related species.

## Introduction

Bloodstream infections caused by *Candida* spp. are associated with high healthcare costs and a mortality rate of 20–60% (Colombo et al., 2006; Nucci et al., 2013; Guinea, 2014; Doi et al., 2016). *Candida albicans* is the primary species involved in these infections. However, infections caused by non-albicans *Candida*, such as *C. parapsilosis*, *C. glabrata*, *C. tropicalis*, *C. krusei*, and *C. guilliermondii*, have been increasing, usually reported in critically ill patients, sometimes with overlapping *C. albicans* infections. In addition, other emerging non-albicans species are described as challenging to properly identify, causing hard to treat infections (Pfaller and Diekema, 2007; Wisplinghoff et al., 2014; Lamoth et al., 2018; Rodrigues et al., 2019; Ahangarkani et al., 2020).

In this context, *Candida haemulonii* is an uncommon and emerging yeast that has been detected in different geographic regions, and it is associated with superficial to deep infections, including chronic otitis media, peritonitis, candidemia, and osteitis, particularly in immunocompromised and neonatal patients (Gargeya et al., 1991; Kim et al., 2009; Hou et al., 2016; Ben-Ami et al., 2017; Arastehfar et al., 2018). In Brazil, species of the *C. haemulonii* complex have also been reported to cause candidemia, associated with infections in critically ill patients (Muro et al., 2012; Ramos et al., 2015; de Almeida et al., 2016).

Classical identification methods are unable to characterize this species, which can be accurately identified using molecular detection methods (Kim et al., 2009; Almeida et al., 2012; Muro et al., 2012; Kumar et al., 2016; Arastehfar et al., 2018). In 1993, *C. haemulonii* species were categorized in genetic groups I and II, which were later reclassified as *C. haemulonii* and *C. haemulonii* var. *vulnera* (group I) and *C. duobushaemulonii* (group II) (Lehmann et al., 1993; Cendejas-Bueno et al., 2012). Recently, two related species of the *C. haemulonii* complex, *C. pseudohaemulonii* and *C. auris*, were described and shown to form a multidrug-resistant (MDR) clade along with *C. lusitaniae* (Muñoz et al., 2018).

*Candida haemulonii* var. *vulnera*, as well as other related species, are commonly resistant to fluconazole and non-susceptible to amphotericin B (AMB). Mechanisms associated with reduced susceptibility to AMB and azoles are often associated with mutations in genes involved in ergosterol biosynthesis, stress responses, and in regulatory regions that promote the overexpression of efflux pumps (Kołaczkowska and Kołaczkowski, 2016; Whaley et al., 2016; Arendrup and Patterson, 2017).

The detection of MDR *Candida* species at the species level is required for better epidemiological surveillance and therapeutic interventions (Cendejas-Bueno et al., 2010; Lockhart et al., 2017b; Mizusawa et al., 2017; Sears and Schwartz, 2017; Spivak and Hanson, 2017; Jeffery-Smith et al., 2018; Taori et al., 2019). Although the molecular basis of the resistance of major *Candida* species to commonly used antifungal agents has been widely described, identifying the genetic determinants of virulence and antifungal resistance in emerging species remains a major challenge. For many of these microorganisms, there is a lack of whole-genome sequencing (WGS) data, leading researchers to use rRNA genes for species classification (Cowen and Steinbach, 2008; Khodadadi et al., 2017). WGS provides a first glimpse of the genomic basis of biological information about the microorganism and may allow further functional studies to elucidate the role of encoded proteins and their metabolic pathways, in order to expand the knowledge about the metabolism, virulence and resistance mechanisms. WGS also enables researchers to uncover phylogenetic relationships between isolates thoroughly (Piskur and Langkjaer, 2004; Binkley et al., 2014; Zoll et al., 2016).

Here we describe the first two genomes of *C. haemulonii* var. *vulnera*, isolated from bloodstream infections of two pediatric patients in a Brazilian hospital and compare them with other members of the MDR clade. Our data allowed the exploration of the phylogenetic context of these isolates, as well as the genomic data that enabled the identification and comparison of virulence and resistance genes. In addition, our orthologous clustering analysis represents a significant contribution to functional protein annotation and a resource for phylogenetic inference of the *Candida* MDR clade.

## Materials and Methods

### Clinical Data and Isolates

The studied clinical isolates were obtained from a tertiary children’s care hospital in the South of Brazil. The yeasts were isolated from blood culture sample of catheter of female patients under 10-years-old. The first case, in December 2009, was a patient with Down Syndrome, diagnosed with cardiopathy (total atrioventricular septal defect); and the second one, in April 2010, was hospitalized with febrile neutropenia after receiving chemotherapy for Ewing’s sarcoma. Patients were treated with a combination of liposomal amphotericin B and fluconazole; and amphotericin B deoxycholate, respectively, exhibiting good clinical recovery. Detailed clinical data sets were previously published (Muro et al., 2012).

### Phenotypic, MALDI-TOF MS Identification and Biofilm Production

Initially, blood cultures were performed using an automated BD Bactec^TM^ 9120 blood culture system (Becton, Dickinson, Franklin Lakes, NJ, United States) and *Candida* spp. were identified using phenotypic and molecular methods. An API 20C AUX system (BioMérieux, Marcy-l’Étoile, France) was used to identify the isolates as *Kodamaea (Pichia) ohmeri*, whereas the Vitek 2^TM^ Compact, YST ID system (BioMérieux) showed inconclusive results for the first isolate (K1) and identified the other (K2) as *C. haemulonii* (Muro et al., 2012). The two strains were confirmed as *C. haemulonii* using matrix-assisted laser desorption ionization mass spectrometry (MALDI-TOF MS) using a Microflex^TM^ LT instrument (Bruker Daltonics, Billerica, MA, United States), and by sequence analysis of the ITS and D1/D2 regions of the rRNA genes (White et al., 1990; Leaw et al., 2006). The isolates were stored in skim milk and frozen at −80°C until processing for further study.

We reviewed the microbiology records including those of the two isolates identified as *C. haemulonii*. Before testing, each isolate was cultured on Sabouraud dextrose agar (Neogen Corporation, Lansing, MI, United States) and CHROMagar *Candida*^TM^ medium (BD Biosciences) for 48 h at 37 and 42°C to ensure their purity, viability, and thermotolerance. These organisms were identified with a Vitek 2^TM^ Compact, YST ID system (BioMérieux) and MALDI-TOF MS, Microflex^TM^ LT instrument (Bruker Daltonics), using controls and the FlexControl^TM^ version 3.4 software according to the manufacturer’s instructions.

Biofilm formation was evaluated as described by Branchini et al. (1994). A loop of microorganisms collected from Sabouraud dextrose agar was inoculated into a polystyrene tube (15 mL Falcon tube) containing 10 mL of Sabouraud dextrose broth supplemented with glucose (final concentration of 8%). The tubes were incubated at 35 ± 2°C for 24 h and the broth was gently aspirated. The tubes were washed thoroughly with phosphate-buffered saline (pH 7.2) and dried. Cells in the dried tubes were stained with 0.1% crystal violet (for 7 min), and excess dye was removed by washing with distilled water. After drying, the tubes were evaluated for biofilm formation. Biofilm formation was considered as positive when a visible film coated the wall and bottom of the tube. The experiments were performed in triplicate and biofilm production was evaluated independently by two different observers.

### Minimum Inhibitory Concentration (MIC)

Antifungal susceptibility testing was performed with Sensititre^TM^ YeastOne^TM^ (Thermo Fisher Scientific, Waltham, MA, United States) using the YO9 AST plate, containing serial drug dilutions of AMB, flucytosine, fluconazole, itraconazole, voriconazole, posaconazole, caspofungin, micafungin, and anidulafungin. Stock inoculum suspensions of the yeasts were obtained from 24-h cultures on Sabouraud dextrose agar at 35°C. The turbidity of each yeast suspension was adjusted to 0.5 McFarland standards and the isolates were tested according to the manufacturer’s instructions. The inoculum was evaluated by colony counting, with *C. parapsilosis* ATCC^®^ 22019 used as the quality control strain. Plates were covered with adhesive seals and the colorimetric MIC endpoints were determined visually after 48 h of incubation at 35 ± 2°C in a non-CO_2_ atmosphere. The interpretative criteria for *in vitro* susceptibility testing of *Candida* spp., set by The Clinical & Laboratory Standards Institute (CLSI) M27-S3 guidelines, were followed, since CLSI M27-S4 contains species-specific clinical breakpoints, except rare species, such as *C. haemulonii* var. *vulnera* (CLSI, 2008, 2017).

### DNA Sequencing

Genomic DNA was extracted using the Wizard^®^ Genomic DNA Purification Kit (Promega, Madison, WI, United States). The Nextera XT DNA system (Illumina, Inc., San Diego, CA, United States) was used to prepare sequencing-ready libraries, which were sequenced using an Illumina MiSeq instrument (paired-end mode, 250 × 2) using the MiSeq Reagent Kit v2 (Illumina, Inc.).

### Assembly, Annotation, and Analysis

We evaluated read quality using fastqc (v.0.11.5)^1^. Trimmomatic (v.0.32) was used to remove adapters and trim and discard reads shorter than 50 base pairs (Bolger et al., 2014). Draft genomes were assembled *de novo* using SPAdes (v.3.5.0) (Bankevich et al., 2012). BUSCO (v.3.0) was used to assess genome assembly completeness (Simão et al., 2015). All genome assemblies were evaluated using QUAST (v.4.6.3), and repetitive sequences were masked using RepeatMasker (v.4.0.7) (Tarailo-Graovac and Chen, 2009; Gurevich et al., 2013). The rRNA and tRNA genes were predicted with RNAmmer (v.1.2.1) and ARAGORN, respectively (Laslett and Canback, 2004; Lagesen et al., 2007). Protein-coding genes were predicted using AUGUSTUS web (Stanke and Morgenstern, 2005). Proteins were annotated using BLAST2GO (v. 5.2.4) against the reference NCBI NR database, Eukaryota taxid: 2759 (minimal query coverage and maximum *E-value* of 40% and 1e-5, respectively) (Conesa et al., 2005). InterPro terms were obtained from InterProScan, which is available from EBI^2^, and converted and merged to Gene Ontology (GO) terms (Quevillon et al., 2005).

### Phylogenetic and Comparative Analysis

The ITS sequences of K1 and K2 were obtained from their respective genomes by identifying the scaffolds in which the ITS regions were located. We performed multiple sequence alignment between each genome and a randomly selected ITS sequence of a *C. haemulonii* species complex isolate with MUMmer (v.4.0.0beta2) (Kurtz et al., 2004). Each identified scaffold (K1 *scaffold_270* for K1 and K2 *scaffold_267* for K2) was aligned with ClustalW (Mega X) to a pair of previously described primers (ITS1 forward: 5’-TCCGTAGGTGAACCTGCGG-3’ and ITS4 reverse: 5’-TCCTCCGCTTATTGATATGC-3’) to identify the start and end of the ITS sequences (White et al., 1990; Kumar et al., 2018). After identifying the ITS sequences, they were extracted with the SAMtools faidx command (v.1.7) (Li et al., 2009).

Phylogenetic analyses were performed with the ITS sequences of the *C. haemulonii* species complex (Supplementary Table S1) and those from K1 and K2 using the unweighted pair group method with arithmetic mean with 1000 bootstrap replicates (Sneath and Sokal, 1973; Felsenstein, 1985). In addition to our two isolates, the analysis included 45 nucleotide sequences: 16 from *C. haemulonii*, 13 from *C. duobushaemulonii*, 12 from *C. haemulonii* var. *vulnera*, two from *C. pseudohaemulonii*, and two from *C. auris* (Chowdhary et al., 2014; Grenfell et al., 2016). The phylogenetic tree was visualized using Interactive Tree Of Life (v.4) (Letunic and Bork, 2016).

Average Nucleotide Identity (ANI) analysis was conducted with pyani (v.0.2.0) based on a whole-genome comparison among K1 and K2, *C. haemulonii* (B11899), *C. pseudohaemulonii* (B12108), *C. duobushaemulonii* (B09383), *C. auris* (6684 and B8441), and *C. lusitaniae* (ATCC 42720 and CBS6939) (Pritchard et al., 2016).

Single nucleotide variants (SNVs) and indel calling among K1, K2, and *C. haemulonii* (B11899) was performed according to GATK Best Practices recommendations (DePristo et al., 2011; Van der Auwera et al., 2013). The pipeline consists of aligning reads from one of the genomes to the other, which serves as a reference (Li, 2011). The alignment was performed using BWA-MEM, and the resulting alignment file was sorted using SAMtools (Li et al., 2009). Duplicated reads were marked using Picard’s MarkDuplicates and sorted with Picard’s SortSam. Variant calling was performed using GATK’s HaplotypeCaller, and the resulting set was normalized with vcflib’s vcfallelicprimitives and filtered (read depth >24 and quality >20) with vcflib’s vcffilter (McKenna et al., 2010). The pipeline was built using Snakemake (Köster and Rahmann, 2018).

We performed orthologous clustering analysis with the web server OrthoVenn2 (Xu et al., 2019). The protein FASTA file containing predicted protein sequences for *C. haemulonii* var. *vulnera* (K1), *C. haemulonii* (B11899), *C. pseudohaemulonii* (B12108), *C. duobushaemulonii* (B09383), *C. auris* (B8441), and *C. lusitaniae* (CBS6939) were used to predict the orthologous gene clusters among the MDR clade. Copy-number variation was assessed using predicted proteins against our database containing genes related to pathogenicity through BLASTp searches with 50 and 60% for similarity and query coverage thresholds, respectively.

Erg11p sequences from our isolates were aligned using MAFFT (v 7.271) (Katoh and Standley, 2013) with sequences from other related species: *C. lusitaniae* (ATCC 42720 and CBS 6936), *C. auris* (6684 and B8441), *C. duobushaemulonii* (B09383), *C. pseudohaemulonii* (B12108), *C haemulonii* (B11899), and *C. albicans* (SC5314).

## Results

### Phenotypic, MALDI-TOF MS Identification and Biofilm Production

The colonies of the two isolates, K1 and K2, when grown on Sabouraud dextrose agar, were white-to-cream-colored and smooth. They grew at 37°C, but not at 42°C, and showed a pink colony color on CHROMagar *Candida*^TM^ medium after incubation for 48 h. One of the isolates (K1) was identified as *C. haemulonii* by Vitek 2^TM^ Compact with 97% certainty, whereas the other (K2) showed inconclusive (low discrimination) results. Considering that commercial phenotypic systems have a limited ability to discriminate *C. haemulonii* complex and related species, our two isolates had their identity confirmed as *C. haemulonii* using MALDI-TOF MS (Ramos et al., 2015). A previous study revealed similarity in the main spectrum profiles of *C. haemulonii* and *C. haemulonii* var. *vulnera* generated by this method, further demonstrating the difficulties of discriminating these microorganisms (Grenfell et al., 2016). Regarding the production of biofilm, both strains were *in vitro* biofilm producers by tube method.

### MIC

The *in vitro* antifungal susceptibility data of *C. haemulonii* var. *vulnera* (*n* = 2) are presented in Table 1. *C. haemulonii* var. *vulnera* was described as a microorganism with high MICs for AMB and for non-susceptible to azoles (Cendejas-Bueno et al., 2012; de Almeida et al., 2016; Kumar et al., 2016). Although there are no interpretive criteria for AMB in the CLSI M27-S3 guidelines, both isolates presented high MIC values for this antifungal agent (≥2.0 μg/mL) (CLSI, 2008). Additionally, both isolates were classified as susceptible dose-dependent to itraconazole, K2 as susceptible dose-dependent to fluconazole, while K1 susceptible to fluconazole, and both as susceptible to echinocandins.

**TABLE 1:** Sensititre^TM^ YeastOne^TM^ antifungal susceptibility profile of *C. haemulonii var. vulnera* (*n* = *2*).

Isolate	MIC μg/mL (CLSI M27-S3 interpretation)								
	**AMB**	**FLU**	**ITC**	**VRC**	**POSA**	**CFG**	**MFG**	**AFG**	**FC**
	
**K1**	8.0 ^(a)^	8.0 (S)	0.50 (S-DD)	0.25 (S)	0.25 ^(b)^	0.25 (S)	0.12 (S)	0.06 (S)	≤0.06 (S)
**K2**	2.0 ^(a)^	16.0 (S-DD)	0.50 (S-DD)	0.25 (S)	0.25 ^(b)^	0.25 (S)	0.12 (S)	0.06 (S)	≤0.06 (S)

*AMB, amphotericin B; FLU, fluconazole; ITC, itraconazole; VRC, voriconazole; POSA, posaconazole; CFG, caspofungin; MFG, micafungin; AFG, anidulafungin; FC, flucytosine; S, susceptible; S-DD, susceptible dose-dependent. ^(a)^ Usually resistant when MIC ≥1 μg/mL, according to M27-A3. ^*(b)*^ Breakpoints not established by CLSI M27-S3.*

### Assembly and Annotation Analysis

We sequenced the genomes of K1 and K2. The draft genomes comprised 350 and 387 contigs, total genome sizes of 13.21 and 13.26 Mb, and an average G + C content of 44%; BUSCO analysis indicated that the assemblies encompassed 94.6 and 94.5%, of the 1,771 genes from the *Saccharomycetales* reference dataset, respectively. A total of 5,484 and 5,508 protein-coding genes were predicted with AUGUSTUS and annotated with Blast2GO (Table 2). Although a major proportion of the *C. haemulonii* var. *vulnera* genome remains uncharacterized, as most of the other *Candida* genomes (Chatterjee et al., 2015); we annotated K1 and K2 genomes using GO. We assigned terms to 4,121 and 4,149 proteins and verified the presence of many catalytic proteins involved in cellular processes, particularly those with hydrolase (23%) and transferase (20%) activity.

**TABLE 2:** Summary description of the *C. haemulonii* var. *vulnera* annotated genome assemblies.

Features	K1	K2
**Genome assembly size**	13,212,126	13,262,707
**Number of contigs**	350	387
**N50**	78,004	75,117
**L50**	53	57
**Largest contig**	392,866	251,044
**Lowest contig**	509	533
**CG content (%)**	45.21	45.20
**Repeat content (%)**	1.16	1.19
**BUSCO completeness assessment (%)^a^**	C: 94.6 D: 0.2 F:2.9 M: 2.5	C: 94.5 D:0.2 F: 2.7 M: 2.8
**Number of rRNAs and tRNAs**	6; 175	6; 173
**Number of predicted genes**	5,479	5,507
**Number of genes blasted^b^**	4,577	4,759
**Number of hypothetical proteins**	3,839	4,014
**Number of genes annotated**	4,121	4,149

*C, complete single-copy; D, duplicated; F, fragmented; M, missing. ^*a*^ Conservation of core genes using BUSCO. ^*b*^*E-value* ≤ 1e-5 and coverage ≥40%.*

**FIGURE 1:**
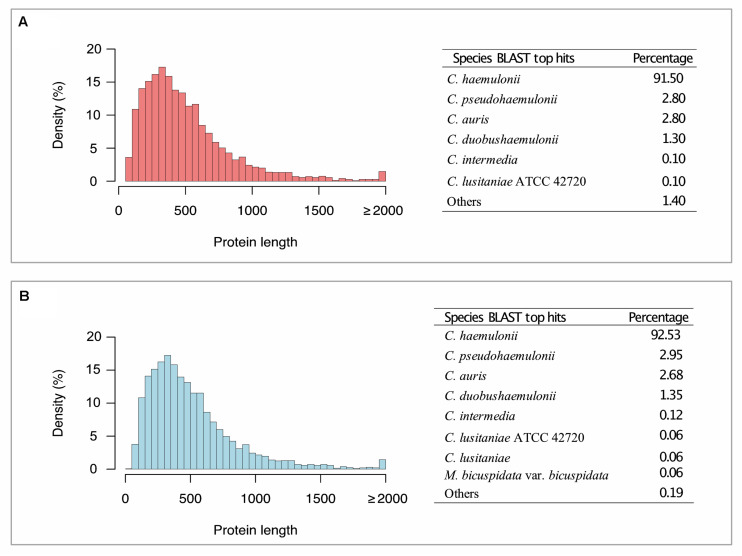
Histogram of coding genes predicted by AUGUSTUS and BLAST top hit species distribution summary. **(A)** *Candida haemulonii* var. *vulnera* K1. **(B)** *Candida haemulonii* var. *vulnera* K2. *Candida haemulonii* var. *vulnera* (K1 and K2) genes annotated against NR database (Eukaryota taxid: 2759), with an *E-value* ≤ 1e-5.

The most frequent species among BLAST hits for K1, and K2 predicted proteins were *C. haemulonii*, *C. pseudohaemulonii*, and *C. auris*, when running against the NCBI nr database (Figure 1). We also annotated genes involved in biofilm formation, multidrug transport, and plasma membrane and cell wall biosynthesis, as well as genes involved in thermotolerance or environmental stress responses, such as *MDR*, *ERG*, *SAP*, *ALS*, *MNN*, *LIP*, *HYR1*, *PMR1*, *VRG4*, *CDR*, *ARB1*, *FKS1*, *FUR1*, *ALG1*, *CHS1*, *CALS11*, *HSFC1B*, and *HSP1*. This whole-genome shotgun project has been deposited at DDBJ/ENA/GenBank under the accession number PRJNA559198 (Biosamples: SAMN12529702 and SAMN12529703).

### Phylogenetic and Comparative Analysis

**FIGURE 2:**
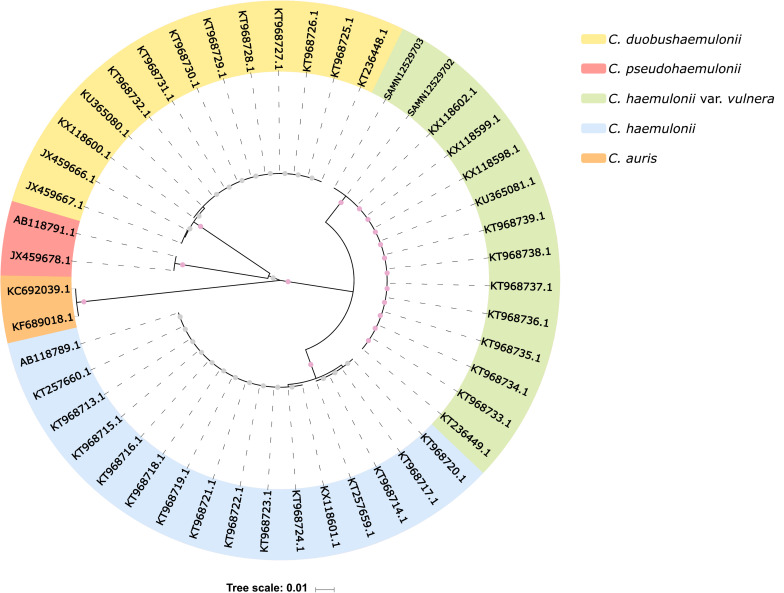
ITS sequence-based phylogenetic tree. Internal dots represent bootstrap values. Gray dots range from 0.60 to 0.70, and pink dots range from 0.99 to 1. SAMN12529703 and SAMN12529702 indicate our isolates (K1 and K2, respectively).

Based on ITS1 and ITS2 sequences from public repositories, we reconstructed the phylogeny of 47 isolates from five different *Candida* species (Supplementary Table S1), including K1 and K2 isolates. This analysis showed that K1 and K2 belong to the *C. haemulonii* var. *vulnera* clade, with 100% bootstrapped support (Figure 2), implying that they have been initially misidentified as *C. haemulonii* by MALDI-TOF MS.

To further evaluate intraspecific variability among *C. haemulonii* complex and related species, we conducted ANI analysis among our isolates and the WGS of *C. haemulonii*, *C. pseudohaemulonii*, *C. duobushaemulonii*, *C. auris*, and *C. lusitaniae* deposited in NCBI. The results confirmed high levels of sequence identity between K1, K2, and *C. haemulonii* (Supplementary Figure S1).

Because this high genomic identity may be associated with repeated genomic regions in eukaryotes, we also searched for SNVs and indel differences among K1, K2, and *C. haemulonii*. Approximately 99.6% of the K2 raw reads were successfully aligned to the K1 genome, and the variant calling between these data resulted in a set of 1,033 SNVs and 154 indels. However, the variant calling of K2, using *C. haemulonii* as a reference resulted in a set of 1,422 SNVs and 1,510 indels, with 99.5% of K2 raw reads successfully aligned to the *C. haemulonii* genome.

Using the OrthoVenn2 web platform (Xu et al., 2019), we performed an orthologous clustering analysis of the predicted proteins in the MDR clade. *Candida lusitaniae* showed a smaller predicted proteome among the included isolates (*C. haemulonii*, *C. haemulonii* var. *vulnera*, *C. duobushaemulonii*, *C. pseudohaemulonii*, and *C. auris*). OrthoVenn2 clustering resulted in a total of 5,625 clusters, 3,849 of which were single-copy clusters.

**FIGURE 3:**
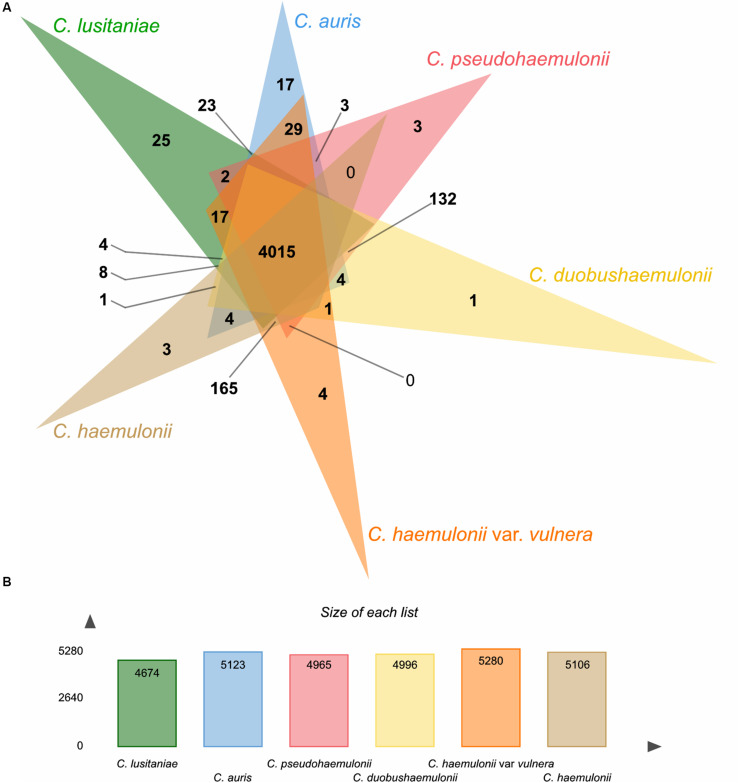
Summary of orthologs analysis using OrthoVenn2. **(A)** Venn diagram showing the distribution of orthologous clusters among *C. haemulonii* var. *vulnera* (K1) and related species. **(B)** Summary of protein data of each species.

A total of 4,015 core clusters were detected (e.g., those with at least one representative from all the six species included), with 24,654 proteins, supporting their conservation in the lineage (Figure 3). *C. lusitaniae* and *C. auris* were the species with the highest number of singleton protein sequences, orthologs for which could not be found in any of the other species. Besides, based on the similarity matrix, *C. auris* genomes formed a higher number of clusters with *C. haemulonii* var. *vulnera* (5,208) which, in turn, formed a higher number of clusters with *C. haemulonii* (5,344), as expected (Supplementary Figure S2).

In addition, 60 proteins from *C. auris* and *C. haemulonii* var. *vulnera* formed 29 clusters that did not include proteins from any other analyzed species. Most of these proteins were assigned the molecular function of ribonucleoprotein complex binding (GO: 0043021) in the nucleus cell region (GO: 0005634).

**FIGURE 4:**
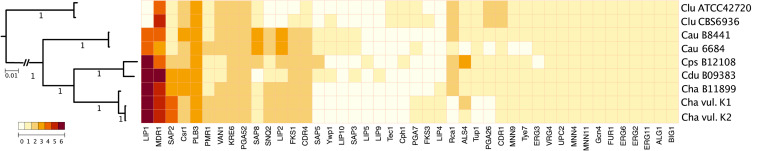
Gene number variation in MDR clade. Maximum likelihood phylogeny using 1737 single-copy core genes based on 1000 replicates among 9 annotated genome assemblies.

To characterize changes in gene content that may play a role in the evolution of MDR clade virulence and multidrug-resistance, we evaluated copy-number variation in genes among *C. haemulonii* var. *vulnera* and seven isolates of related species (Figure 4). Although many gene families involved in the pathogenesis of diseases caused by these species are present in similar numbers, some differences were observed, such as the expansion of lipases (particularly *LIP1*) shared by *C. auris*, *C. pseudohaemulonii*, *C. duobushaemulonii*, *C haemulonii*, and *C. haemulonii* var. *vulnera*, but not *C. lusitaniae*.

We identified, among species of the MDR clade, including our isolates, a greater number of ABC (ATP-binding cassette) transporter genes involved in clinical resistance to fluconazole and other toxic compounds, particularly the multidrug-resistant transporter 1 (*MDR1*). Additionally, *C. lusitaniae* was the only species that showed an increased copy-number of *Candida* drug resistance protein 1 (*CDR1*).

**FIGURE 5:**
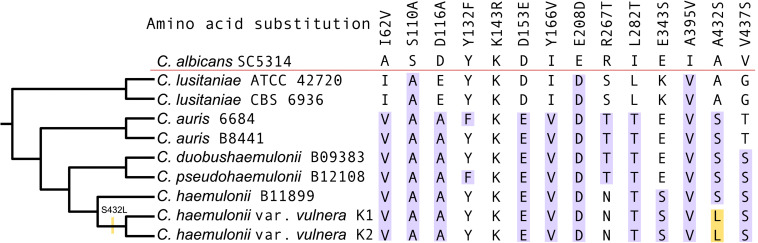
Multiple sequence alignment of the Erg11p from K1, K2, and other *Candida* species. Highlighted in blue are the previously described amino acid substitutions identified among the isolates and in yellow the apomorphy.

Alignment of the Erg11p amino acid sequences of K1 and K2 with MDR clade isolates and *C. albicans* (sensitive to polyene and azole) revealed ten amino acid substitutions previously reported as associated with azole resistance in *Candida*. All substitutions except E343S, V437S, A395V, and Y166V have been previously listed into three hotspot regions at amino acids 105–165, 266–287, and 405–488 of *C. albicans* Erg11p (Morio et al., 2010; Silva et al., 2016). However, the hotspot mutations Y132F and K143R, often found in resistant strains, were not identified in the two isolates of *C. haemulonii* var. *vulnera* described here (Figure 5; Healey et al., 2018).

An apomorphy of the K1 and K2 isolates was observed at position 432 (A432L) in Erg11p, although serine in the position 432 was most likely the ancestral state of the MDR clade (Figure 5). The substitution of serine by leucine in the K1 and K2 isolates represented the reversal of e apolarity conferred by alanine in azole-sensitive members of *Candida*, such as *C. albicans* and *C. lusitaniae*.

## Discussion

The occurrence of candidiasis, caused by non-albicans species, is increasing. Although the reasons are still unclear, the use of fluconazole prophylaxis, the growing number of patients who are at risk of invasive fungal infections and advances in microbiological diagnosis, may have contributed (Sardi et al., 2013; Lee et al., 2018).

The emergence of non-albicans species has reinforced the importance of improving microbiological laboratory procedures, in particular concerning the adoption of molecular methods to overcome the limitation of classical methods in the identification of these opportunistic microorganisms (Lockhart et al., 2017a). Although MALDI-TOF MS is fast and accurate for yeast identification, it shows variable performance in the discrimination of *C. haemulonii* complex isolates and related species (Kathuria et al., 2015; Grenfell et al., 2016; Taverna et al., 2019). The two isolates reported here were identified as *C. haemulonii sensu stricto* using MALDI-TOF MS, while ITS1 and ITS2 analysis, obtained from the assembled genomes, identified the isolates as *C. haemulonii* var. *vulnera* (Supplementary Table S1).

Members of the *C. haemulonii* species complex are emerging yeasts associated with bloodstream and other invasive infections (Ruan et al., 2010). They are phylogenetically related to *C. auris*, the first global emerging yeast pathogen with potential for nosocomial transmission (Jeffery-Smith et al., 2018). Although *C. auris* is recognized as a virulent yeast, it exhibits high sequence divergence compared to *Candida* species commonly involved in human infections, such as *C. albicans*, *C. tropicalis*, *C. parapsilosis*, and *C. glabrata*. *C. auris* is more closely related to the *C. haemulonii* complex species, such as *C. pseudohaemulonii* and *C. lusitania*e, which are less virulent but resistant to antifungals (Jeffery-Smith et al., 2018).

Although not yet reported in Brazil, *C. auris* has been identified in more than 30 countries. Complete genomic data revealed four distinct geographic clades: South Asian (I), East Asian (II), African (III), and South American (IV) (Lockhart et al., 2017b; Muñoz et al., 2018). More recently, a new clade, Iran (V), has also been proposed (Chow et al., 2019). Nevertheless, the origin of *C. auris* remains uncertain (Jackson et al., 2019). The generation and analysis of WGS data presented here can be used to explore the population structure and help to understand the species origin of MDR clade, and monitoring the global dissemination of them (Chybowska et al., 2020).

Here, we report the first WGS, assembly, and annotation of two *C. haemulonii* var. *vulnera* isolated from pediatric patients with candidemia in a tertiary pediatric hospital in the South of Brazil. The genomes are approximately 13.21 Mb and contain more than 5,400 predicted genes, similar to what has been previously described for other *Candida* species (Durrens et al., 2017; Chow et al., 2018a, b; Mohd Tap et al., 2018). ANI analysis showed high identity among K1 and K2 and *C. haemulonii* B11899 (99%). Comparison of *C. haemulonii* var. *vulnera* genomes to other related species showed an identity of 77% compared with *C. duobushaemulonii* or *C. pseudohaemulonii*, 75% with *C. auris*, and 72% with *C. lusitaniae* (Supplementary Figure S1).

A concatenated alignment of 1737 single-copy core genes from MDR clade members was used for the maximum likelihood tree inference. As expected, the data supported that *C. haemulonii* and *C. haemulonii* var. *vulnera* were closely related to each other, followed by *C. duobushaemulonii* and *C. pseudohaemulonii* that formed a sister group to *C. haemulonii* and *C. auris* as more divergent species. In turn, *C. lusitaniae* is a basally branching member of this group, consistent with previous phylogenetic analyses (Figure 4; Muñoz et al., 2018).

The pathogenicity of *Candida* spp. is attributed to virulence factors that allow evasion from the host’s defenses, enables adherence to surfaces, biofilm formation, alteration of cellular structure, and production of hydrolytic enzymes (Kumar and Menon, 2006; Ramage et al., 2009). A complex array of intracellular mechanisms and external factors influence biofilm formation and architecture in *Candida* species (Oh et al., 2011; Cavalheiro and Teixeira, 2018) as well as our two isolates, species from the *C. haemulonii* complex that have been reported to produce biofilm (Oh et al., 2011; Cendejas-Bueno et al., 2012; Muro et al., 2012; Ramos et al., 2017).

Detecting biofilm production is important, since biofilms provide greater resistance to antifungal agents and host’s defenses, contributing to the persistence of the infection. When detected on abiotic surfaces, such as catheters, the medical device should be removed (Tumbarello et al., 2012; Cavalheiro and Teixeira, 2018). Comparative genomics analysis revealed that, among the sequenced *Candida* species, *C. haemulonii* var. *vulnera* is closest to the previously characterized *C. haemulonii* and shares significant virulence attributes with the other members of the MDR clade, such as multiple drug transporter proteins, proteases and lipases.

Currently, invasive candidiasis is treated with three main classes of drugs: polyene antifungal agents, azoles, and echinocandins (Ben-Ami, 2018). Azole antifungals are important to treat *Candida* spp. infections, but the emergence of resistance to azoles and polyenes often culminates in treatment failure (Whaley et al., 2016; Lotfali et al., 2017). Voriconazole is associated with more adverse effects and drug interactions than fluconazole, and offers few advantages in candidiasis treatment, proving infection treatment by species intrinsically resistant to fluconazole (*C. krusei*) useful. Echinocandins are highly potent and safe antifungals for clinical use, against a wide variety of *Candida* species, including tolerant or azole-resistant ones (Ben-Ami, 2018).

Considering the few classes of antifungal agents available for the invasive candidiasis, the isolates described in this study were considered as MDR, because they were not susceptible to ≥1 agent (AMB, fluconazole and itraconazole) in ≥2 classes of drugs (polyene and azole) (Arendrup et al., 2017). Our results reinforced the importance of accurate identification and proper antifungal susceptibility testing to guide therapeutic strategies, instead of using AMB and azoles empirically.

Multiple mechanisms of resistance to azoles have been described for *Candida* species, including mutations in ergosterol biosynthesis genes (mainly resulting in increased expression of *ERG11*), overexpression of drug efflux pumps (e.g., *CDR1*, *CDR2*, and *MDR1*), and function-enhancing mutations in transcription factors (e.g., *TAC1* and *MRR1*) that enhance their expression (Flowers et al., 2015; Whaley et al., 2016). Mechanisms of polyene resistance are less well studied than those of azoles resistance. However, the main mechanism of resistance to AMB involves ergosterol content reduction in the cell membrane (target abundance). Mutations in ergosterol biosynthesis genes (mainly *ERG11* and *ERG3*) and biofilm formation can confer azole-polyene cross-resistance. Further, treatment with azoles that reduces cellular sterol concentrations can also confer polyene resistance (Lotfali et al., 2017; Perlin et al., 2017). Here we reported the presence of genes involved in biofilm formation, resistance genes and amino acid substitutions in Erg11p related to the increased MIC of azole and polyene antifungal agents in *Candida* spp.

The results presented here can be used to study fungal biology and virulence, as well as to understand the phylogenetic relationships between MDR *Candida* better, and to identify genomic regions associated with species-specific phenotypes.

## Data Availability Statement

The datasets presented in this study can be found in online repositories. The names of the repository/repositories and accession number(s) can be found at: https://www.ncbi.nlm.nih.gov/bioproject/?term=PRJNA559198.

## Ethics Statement

The studies involving human participants were reviewed and approved by HPP Institutional Review Board (IRB) number: 660-08/2008. Written informed consent to participate in this study was provided by the participants’ legal guardian/next of kin.

## Author Contributions

LD-C, TV, LR, and HP-A conceived of the study. LD-C and TV supervised and managed the project. LR, RG, HP-A, AV, PP, RN, and RS performed research and data analysis. LR wrote the manuscript which was read, edited and approved by all authors. All authors contributed to the article and approved the submitted version.

## Conflict of Interest

The authors declare that the research was conducted in the absence of any commercial or financial relationships that could be construed as a potential conflict of interest.

## Footnotes

1 https://www.bioinformatics.babraham.ac.uk/projects/fastqc/

2 http://www.ebi.ac.uk/interpro/
