# Supplementary material for: First Genome Sequences of Two Multidrug-Resistant Candida haemulonii var. vulnera Isolates From Pediatric Patients With Candidemia
Source: Front Microbiol. 2020 Jul 3;11:1535. doi: 10.3389/fmicb.2020.01535 (PMC7350289; doi:10.3389/fmicb.2020.01535)
Supplement: Supplementary file 1 [file Data_Sheet_1.pdf]

S1 TABLE. Isolates of the *Candida haemulonii* complex included in the phylogenetic analysis of internal transcribed spacers (ITS1 and ITS2) sequences

Isolate	GenBank accession no.	Species
HCFMUSP01	KT236448.1	*C. duobushaemulonii*
HCFMUSP02	KT968725.1	*C. duobushaemulonii*
HCFMUSP03	KT236449.1	*C. haemulonii* var. *vulnera*
HCFMUSP04	KT968726.1	*C. duobushaemulonii*
HCFMUSP05	KT968713.1	*C. haemulonii*
HCFMUSP06	KT968733.1	*C. haemulonii* var. *vulnera*
HCFMUSP07	KT968734.1	*C. haemulonii* var. *vulnera*
HCFMUSP08	KT968735.1	*C. haemulonii* var. *vulnera*
HCFMUSP09	KT968714.1	*C. haemulonii*
HCFMUSP10	KT257659.1	*C. haemulonii*
HCFMUSP11	KT968727.1	*C. duobushaemulonii*
HCFMUSP12	KT968715.1	*C. haemulonii*
HCFMUSP13	KT968716.1	*C. haemulonii*
HCFMUSP14	KT968728.1	*C. duobushaemulonii*
HCFMUSP15	KT968729.1	*C. duobushaemulonii*
HCFMUSP16	KT968730.1	*C. duobushaemulonii*
HCFMUSP17	KT968717.1	*C. haemulonii*
HCFMUSP18	KT968731.1	*C. duobushaemulonii*
HCFMUSP19	KT968718.1	*C. haemulonii*
HCFMUSP20	KT968732.1	*C. duobushaemulonii*
HCFMUSP21	KT968719.1	*C. haemulonii*
HCFMUSP22	KT968720.1	*C. haemulonii*
HCFMUSP23	KT968721.1	*C. haemulonii*
HCFMUSP24	KT968736.1	*C. haemulonii* var. *vulnera*
HCFMUSP25	KT968737.1	*C. haemulonii* var. *vulnera*
HCFMUSP26	KT968722.1	*C. haemulonii*
HCFMUSP27	KT257660.1	*C. haemulonii*
HCFMUSP28	KT968723.1	*C. haemulonii*
HCFMUSP29	KT968738.1	*C. haemulonii* var. *vulnera*
HCFMUSP30	KT968724.1	*C. haemulonii*
HCFMUSP31	KT968739.1	*C. haemulonii* var. *vulnera*
HCFMUSP32	KU365080.1	*C. duobushaemulonii*
HCFMUSP33	KU365081.1	*C. haemulonii* var. *vulnera*
IMT-USP01	KX118598.1	*C. haemulonii* var. *vulnera*

HCFMUSP34	KX118599.1	*C. haemulonii* var. *vulnera*
HCFMUSP35	KX118600.1	*C. duobushaemulonii*
HCFMUSP36	KX118601.1	*C. haemulonii*
HCFMUSP37	KX118602.1	*C. haemulonii* var. *vulnera*
CBS5149T	AB118789.1	*C. haemulonii*
CBS7798T	JX459666.1	*C. duobushaemulonii*
CBS7799	JX459667.1	*C. duobushaemulonii*
CBS10004	AB118791.1	*C. pseudohaemulonii*
KCTC17807	JX459678.1	*C. pseudohaemulonii*
CBS12883	KF689018.1	*C. auris*
CBS12766	KC692039.1	*C. auris*

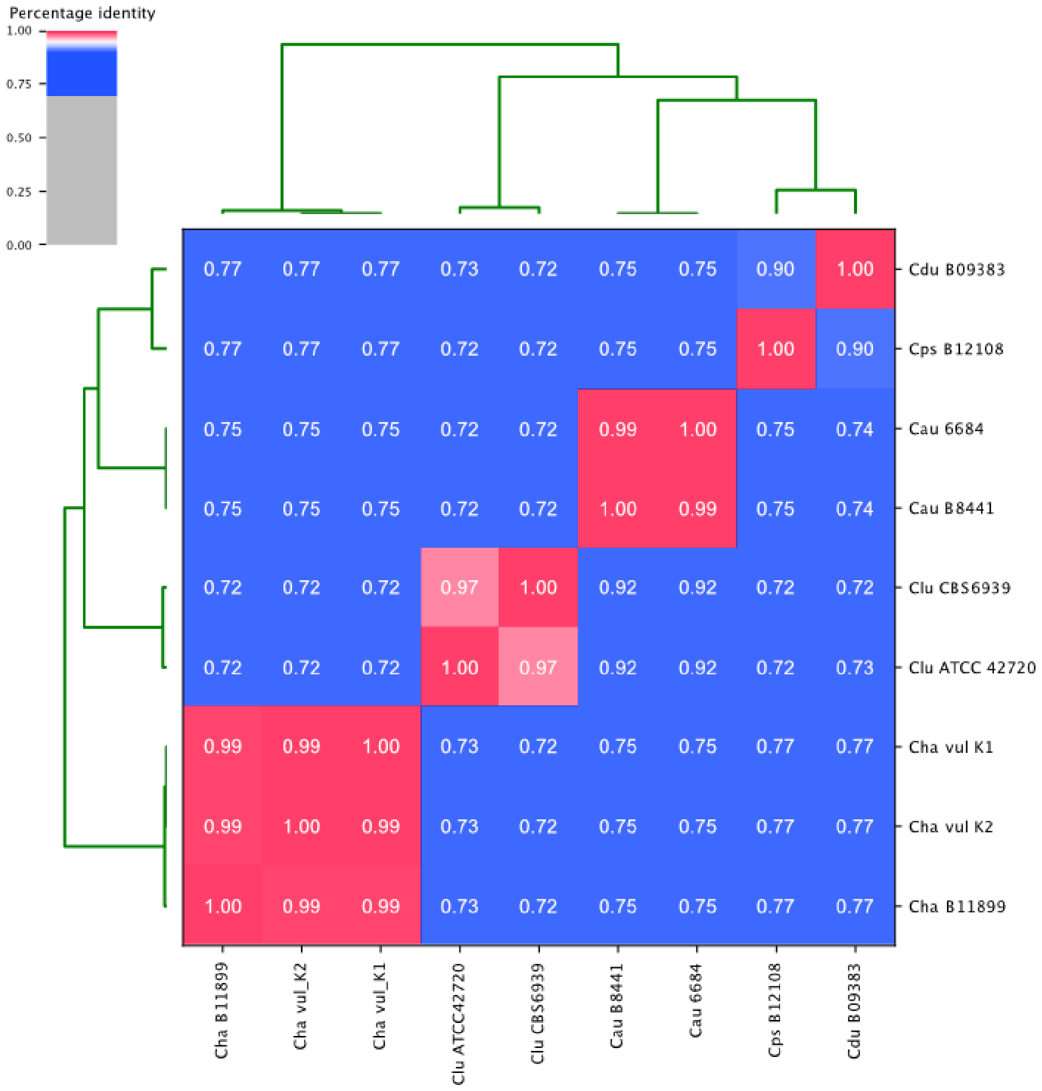

FIGURE S1. ANI analysis between *C. haemulonii* B11899, *C. haemulonii* var. *vulnera* (K2 and K1), *C. lusitaniae* (ATCC 427720 and CBS6939), *C. auris* (B8441 and6684), *C. pseudohaemulonii* (B12108) and *C. duobushaemulonii* (B09383).

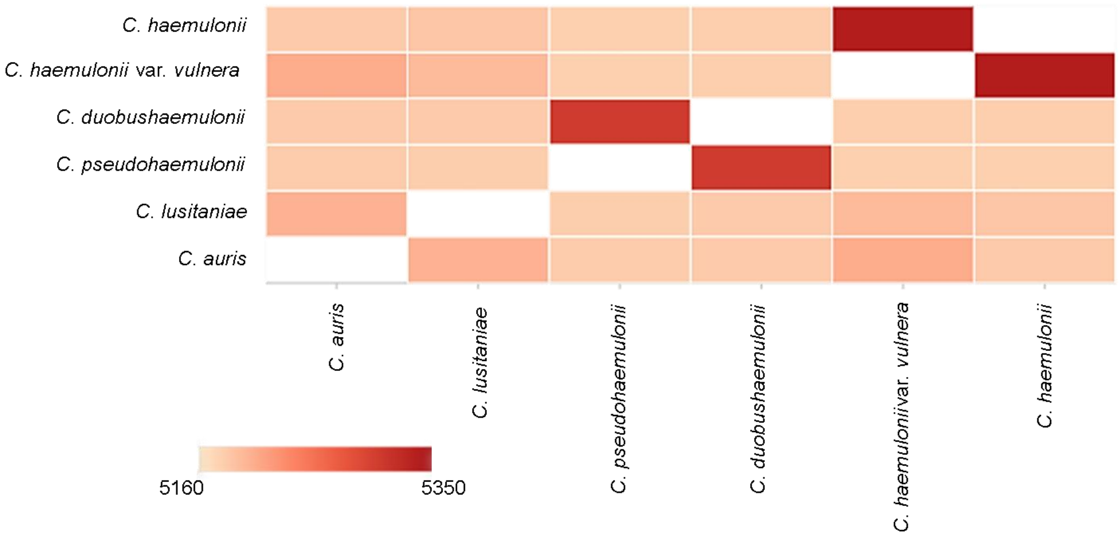

FIGURE S2. Heat map to visualize the intensity of overlapping clusters for the species in a pairwise fashion.
